# The effect of improving task representativeness on capturing nurses’ risk assessment judgements: a comparison of written case simulations and physical simulations

**DOI:** 10.1186/1472-6947-13-62

**Published:** 2013-05-30

**Authors:** Huiqin Yang, Carl Thompson, Robert M Hamm, Martin Bland, Alison Foster

**Affiliations:** 1 Centre for Reviews and Dissemination, University of York, York YO10 5DD, UK; 2 Department of Health Sciences, University of York, York YO10 5DD, UK; 3 Department of Family and Preventive Medicine, University of Oklahoma, Oklahoma, OK 73104, USA

**Keywords:** Written case simulation, Physical simulation, Representative design, Clinical judgement analysis, Risk assessment, Lens model equation, Logistic regression, Clinical vignettes

## Abstract

**Background:**

The validity of studies describing clinicians’ judgements based on their responses to paper cases is questionable, because - commonly used - paper case simulations only partly reflect real clinical environments. In this study we test whether paper case simulations evoke similar risk assessment judgements to the more realistic simulated patients used in high fidelity physical simulations.

**Methods:**

97 nurses (34 experienced nurses and 63 student nurses) made dichotomous assessments of risk of acute deterioration on the same 25 simulated scenarios in both paper case and physical simulation settings. Scenarios were generated from real patient cases. Measures of judgement ‘ecology’ were derived from the same case records. The relationship between nurses’ judgements, actual patient outcomes (i.e. ecological criteria), and patient characteristics were described using the methodology of judgement analysis. Logistic regression models were constructed to calculate Lens Model Equation parameters. Parameters were then compared between the modeled paper-case and physical-simulation judgements.

**Results:**

Participants had significantly less achievement (r_a_) judging physical simulations than when judging paper cases. They used less modelable knowledge (G) with physical simulations than with paper cases, while retaining similar cognitive control and consistency on repeated patients. Respiration rate, the most important cue for predicting patient risk in the ecological model, was weighted most heavily by participants.

**Conclusions:**

To the extent that accuracy in judgement analysis studies is a function of task representativeness, improving task representativeness via high fidelity physical simulations resulted in lower judgement performance in risk assessments amongst nurses when compared to paper case simulations. Lens Model statistics could prove useful when comparing different options for the design of simulations used in clinical judgement analysis. The approach outlined may be of value to those designing and evaluating clinical simulations as part of education and training strategies aimed at improving clinical judgement and reasoning.

## Background

Judgement analysis (JA) has a long history as a means of examining the judgement strategies and performance of clinicians. The theoretical basis for judgement analysis is the Lens Model proposed by Brunswik [1] and developed by Hammond et al. [2-4]. Applied to clinical judgement, the Lens Model describes an individual clinician’s judgements and the clinical environment using comparable models [5], providing a rigorous conceptual and empirical approach for understanding the task, a clinician’s judgements and unpacking the accuracy of those judgements.

Assume we have data about a set of patients sampled from a clinical setting (an ecology). Assume also, we have information on their clinical features and know what happened to them (a criterion, such as death or an adverse event). A clinician’s predictions of that criterion are based on the same clinical features. The Lens Model Equation describes the reliability and the accuracy of the clinician’s judgement via five decompositional concepts [6]: predictability (Re); cognitive control (Rs); achievement (r_a_); policy matching (G); and unmodeled knowledge (C). Predictability (Re) reflects the degree to which a model predicts the value of the ecological criterion from the clinical features. Cognitive control (Rs) reflects how well a similar model predicts the clinician’s judgements based on the same features; Rs examines the consistency with which the clinician applies a policy to their judgments. Achievement (r_a_) measures the correspondence between the person’s judgements and the ecological criterion, i.e., the judgement accuracy. Policy matching (G) reflects the degree to which the clinician’s judgement model captures the modeled component of the ecology. Unmodeled knowledge (C) measures the degree to which the residuals from the model of the clinician’s judgements reflect the unmodeled (residual) components of the ecology.

Judgement analysis is a powerful decompositional tool but using it requires the researcher to overcome a significant methodological challenge: its generalisability depends on how well the tasks used as the basis for modeling a person’s judgement represent the conditions in which such judgements are usually made [7-9]. Clinical judgement analysts typically use paper-based scenarios. While paper cases have the advantage of ease of administration [10,11], their ability to evoke judgements that are similar to clinicians’ responses to actual judgement situations is questionable [12-14]. Format also shapes the cognitive effort invested in processing a task [13]; reflected, for example, in the amount of information subjects use [15-17]. Clinical practice presents large numbers of cues perceptually and simultaneously, which may induce intuitive judgement [3,18]. Collecting visual and other perceptual/sensory information is a crucial component of the clinical judgement process in clinicians. Using non-sensory cues, or sensory cues converted to a written format (as proxies for the “real thing”), e.g., paper cases rather than physical patients to present information to clinicians, may not adequately evoke the cognition used in clinical environments. Thus, paper case simulations may threaten the validity of the results of a judgement analysis.

In contrast, high fidelity physical simulations (for example, using computerized patient simulators) make use of more perceptual cues and so may generate more representative (and thus generalizable) judgement models. Computerized patient simulators have the potential to enhance the fidelity of simulations and foster task representativeness in JA. Computerized patient simulators are able to recreate observable physiological information cues such as audible heart and breath sounds along with displays of common physiological data on the bedside monitor [19-21].

Given the limitations of traditional paper-based approaches, we explored the potential of high fidelity physical simulations for examining nurses’ risk assessment judgements identifying patients at risk of deterioration in acute care settings. This is a judgement that health care professionals, including nurses, do not always perform optimally; physiological deterioration is often unrecognised, inadequately and/or inappropriately treated [22-24]. Early recognition of important changes in physiological parameters is critical if ‘failure to rescue’ and/or a critical event such as cardiac or respiratory arrest is to be avoided [25]. In using risk assessment as the “test bed” for our modeling, we aimed to test the hypothesis that high fidelity physical simulations - by realistically simulating naturally occurring clinical information - can prompt more realistic nurse judgements than paper cases. We can test this hypothesis by comparing the Lens Model Equation parameters derived from the paper cases and the high fidelity physical simulations.

## Methods

### Study design

Each participant assessed the risk of a set of described patients in two phases, with paper cases and with high fidelity physical simulations produced by a computerized patient simulator (Laerdal ™SimMan, Stavanger, Norway, http://www.laerdal.com). In each phase, we used the ‘double system’ approach to judgement analysis [26]. This design investigates judgement accuracy by explicitly comparing participants’ judgements with the ecological criteria. The judgement accuracy is represented by the correlation coefficient between a participant’s judgements (e.g. “at risk”) and ecological criteria (“presence of a critical event”). To minimize any maturation effect [27] due to the time gap between the two phases, the physical simulation experiment was conducted within 5 to 7 days after phase one’s paper cases had been completed.

### Construction of clinical scenarios

Real patient cases were sampled from a data set of emergency admissions (n = 673) collected prospectively in the Medical Admissions Unit of a single NHS District General Hospital during March 2000 by Subbe et al. [28]. Although the original dataset from which the scenarios were constructed was collected in 2000, there is little reason to believe that the relationship between patients’ physiological parameters and outcomes has changed significantly. Certainly, the judgement of “risk of deterioration” was, and remains, an important one. The ecological criterion (‘at risk’ or ‘not at risk’) was determined from the patient dataset; classifying patients as ‘at risk’ if later they died, were admitted to Intensive Care Unit (ICU)/High Dependency Unit (HDU) or experienced cardiopulmonary resuscitation. A stratified random sample based on whether the patients had an acute deterioration or not was used to select clinical scenarios from the dataset records for use in the judgement task. We used 5 clinical cues in the construction of clinical scenarios for assessing a patient at risk of acute deterioration: systolic blood pressure, heart rate, respiratory rate (RR), temperature, and levels of consciousness. These 5 cues are identified as valid by a NICE clinical guideline [29], and all are widely used in rapid assessments of risks in critical care patients [30].

### Scenario sample size

Ten ‘at risk’ cases and 10 ‘not at risk’ cases were sampled, using a random number generator. Participants were shown 25 scenarios, including 5 repeated cases. The repetitions (3 ‘at risk’ patients and 2 ‘not at risk’) were included in the pool of 25 clinical scenarios to enable consistency checking. Cooksey [26] recommends a ratio of 5 to 10 scenarios per cue in judgement analysis as a desirable basis for sample size estimation. Our sample was not adequate for analysis of all 5 cues because: the cues were highly intercorrelated; Cooksey’s rule of thumb arguably does not apply to the 5 repeated cases; and the rule is proposed for multiple linear, rather than multiple logistic, regression.

### Characteristics of cues

Patient features for sampled cases are given in Tables 1 and 2. The intercorrelations among the 5 cues and the “at risk” criterion for the sampled cases showed that six pairs of cues were significantly correlated (0.475 to 0.616). Four cue intercorrelations in the patient cases were large (r > =0.50) and 2 were moderate (r > =0.30) [31].

**Table 1:** Distributional characteristics of cues: the continuous cue variables for the patient cases (N = 20)

**Cues**	**Mean (SD)**	**Minimum**	**Median**	**Maximum**	**Skewness**	**Kurtosis**
Systolic BP	127 (31)	78	127	216	0.97	2.75
Heart rate	93 (22)	56	93	128	0.12	−1.25
Respiration rate	24.2 (7.9)	12	22	40	0.97	0.12
Temperature	37.08 (0.90)	35.60	37.00	39.60	1.33	2.85

**Table 2:** Distributional characteristics of cues: the categorical cue of consciousness level for the patient cases (N = 20)

**Levels of consciousness**	**Frequency (percentage)**
Alert	13 (65%)
Reacting to voice	5 (25%)
Reacting to pain	2 (10%)

Table 3 illustrates cue multicollinearity using the tolerance statistic [32]. If the tolerance is small, the variable is almost a perfect linear combination of the other cues, and should be excluded from regression equations [32]. Rules of thumb are variously that tolerance under 0.10, or 0.20 [32], or 0.40, is worrisome [33], and that the cutoff should be higher when the sample is small. These considerations suggest that multicollinearity between cues in the sampled patients may affect the accuracy of regression models.

**Table 3:** Multicollinearity diagnostics (tolerance) for the patient cases (N = 20)

**Cues**	**Tolerance**
Consciousness	0.18
Systolic BP	0.25
Temperature	0.43
Respiration rate	0.45
Heart rate	0.47

Note: Tolerance is 1 – R^2^, the complement of the proportion of variance explained in a multiple linear regression predicting the cue from all the other cues.

### Participant sample size

We used previous research [34] to estimate the standard deviation (0.14) of participants’ mean r_a_ of 0.43 for risk assessments in paper scenarios. These correlations were normalized using Fisher’s Z transformation [35]. Using the method suggested by Bland [35], and assuming that the correlation coefficient between paired measurements of participants in paper case and physical simulation scenarios is 0.8, the detectable difference in mean correlation coefficients (r_a_) would be 0.02. This relatively small difference would be detected with power 0.90 and a significance level of 0.05 (two-sided) with 90 people. An estimated sample size of 90 participants was therefore adequate. Given the higher recruitment costs associated with experienced nurses compared to student nurses, a ratio of 2 students for every experienced nurse was used as the basis for recruitment. Using moderately unequal independent samples has little compromising effect on statistical power [36].

### Sampling participants and ethical approval

We sampled 34 experienced nurses from the critical & acute care registered nurse population in North Yorkshire hospitals and 63 student nurses from the undergraduate (2nd and 3rd year) student nurse population in the Department of Health Sciences at the University of York, UK. Each participant received a letter and research information sheet inviting them to participate. Ethical approval was obtained from the Health Sciences Research Governance Committee of the University of York, UK. All nurses completed a written informed consent document.

### Presentation of clinical scenarios

#### Paper scenarios

For the paper cases, a clinical vignette booklet was designed to present background clinical information for a generic emergency patient, and then 25 clinical scenarios containing varying cue values for specific patients (see Additional file 1). Clinical cues were presented in natural units such as mmHg (for systolic blood pressure), beats per minute (heart rate), breath per minute (respiratory rate) and degree Celsius (°C) (temperature). Patient consciousness was represented using three levels: alert (A), reacting to voice (V), and reacting to pain (P). The format and content were approved by a critical care specialist nurse with more than 10 years specialist experience.

#### High fidelity physical simulation

**Figure 1:**
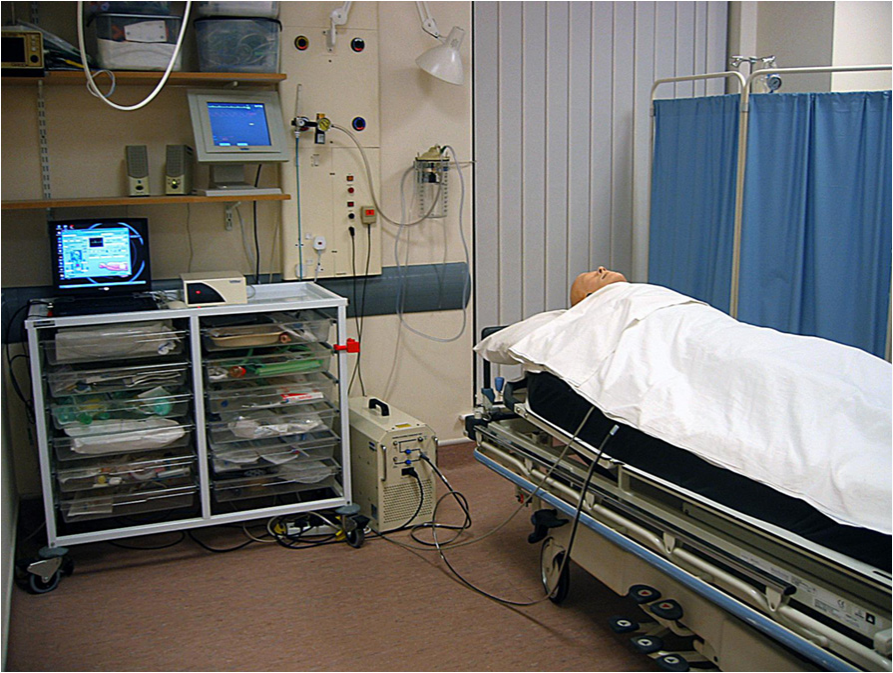
High fidelity physical simulation setting.

The clinical simulation lab in the Department of Health Sciences of the University of York, UK was used to recreate an emergency medical admission unit environment (see Figure 1). The computerized patient simulator (Laerdal ™SimMan) was used to recreate the same 25 clinical scenarios used in the paper case scenarios. The same patient background clinical information was outlined on a whiteboard in the simulated high dependency room.

The computerized patient simulator was used to represent the same physiological cues used in the paper cases: systolic blood pressure, heart rate, respiratory rate, temperature, and consciousness. The numerical values of the first 4 cues were presented in the bedside monitor, displayed in a way that is identical to real practice settings (see Figure 1). Respiration sounds were emitted by the computerized patient simulator, synchronized with the bedside monitor. Consciousness level was represented by different vocalizations. The consciousness level of alert was represented by the vocalization, “Oh, I feel really ill.” Reacting to voice was represented by, “Ouch! Where is my wife? Get off me! Get off Me!” Reacting to pain was represented by a moan. The development of the vocal sounds to denote levels of consciousness was undertaken with the help of the nurse specialist in critical care and simulation. Prior to making the judgements, all participants were informed what level of consciousness each vocal sound represented. They were instructed to use all the information, including the vital signs shown on the bedside monitor as well as the vocalized consciousness level and breath sounds of the computerized patient simulator. Participants were given the same amount of time for both paper-based and physical simulation scenarios.

### Capturing judgements

**Figure 2:**
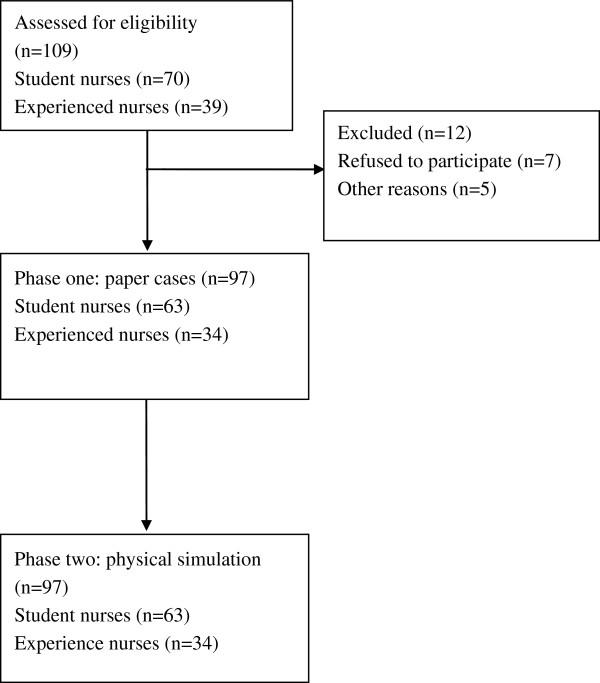
Experimental design and flow of participants through the study.

Participants were asked to judge whether the simulated patient is at risk of acute deterioration (yes ‘at risk’/no ‘not at risk’) for each patient scenario on a data collection sheet in both paper case simulation and high fidelity physical simulation conditions. In paper case simulations, participants were invited to a classroom in the University of York to complete the clinical vignette questionnaire. In high fidelity physical simulations, three to five participants at a time were invited to conduct the experiment simultaneously. A researcher was responsible for giving an introduction to the experiment. In both conditions, all the participants were told to make a ‘yes’ judgement on risk of acute deterioration if he/she judged that the patient would later die, experience cardiopulmonary arrest or be admitted to ICU/HDU for intensive interventions. Prior to the experiment, all the participants were fully orientated to the simulation facilities and all participants confirmed that they were familiar with the judgement task environment based on their previous clinical or learning experience; all participants conformed that they clearly understood the risk assessment judgement task required of them. Nurses were encouraged to make their judgements in the same way they would in real practice. Participants independently made all their judgments without discussion during the physical simulation sessions. The design of this study and flow of the participants through it is presented in Figure 2. The data were collected in 2009 with primary and supplementary analyses conducted between 2009 and 2012.

### Data analysis

#### Analysis of Lens Model Equation parameters

Logistic regression models of the relationship between the outcome measure judged by the nurse and cues in the scenarios were constructed. The outcome variable was the participant’s dichotomous judgements (yes/no) while the predictors were the information cues in scenarios. Logistic regression analysis was also used to derive a model predicting the ecological criterion (yes/no) with the predictors being the same cues as in the judgement model. Model predictions are the probabilities of the “yes” category.

The statistics of the Logistic Lens Model Equation were examined using the following formula [37]:

(1) $$\begin{array}{ll} r_{a}= & G\frac{\sigma_{{\tilde{Y}}_{e}}}{\sigma_{Y_{e}}}\frac{\sigma_{{\tilde{Y}}_{s}}}{\sigma_{Y_{s}}}+C_{1}\frac{\sigma_{{\tilde{Z}}_{e}}}{\sigma_{Y_{e}}}\frac{\sigma_{{\tilde{Z}}_{s}}}{\sigma_{Y_{s}}}+C_{2}\frac{\sigma_{{\tilde{Y}}_{e}}}{\sigma_{Y_{e}}}\frac{\sigma_{{\tilde{Z}}_{s}}}{\sigma_{Y_{s}}}\\ & +C_{3}\frac{\sigma_{{\tilde{Z}}_{e}}}{\sigma_{Y_{e}}}\frac{\sigma_{{\tilde{Y}}_{s}}}{\sigma_{Y_{s}}} \end{array}$$

In the first section, the term

(2) $$G=r_{{\tilde{Y}}_{e}{\tilde{Y}}_{s}}$$

is the correlation between the predicted judgement of the participant, the estimated probability the participant assesses the patient as “at risk,” and the predicted criterion of the ecology, the estimated probability the patient was “at risk”. The portion of the equation

(3) $$C_{1}=r_{{\tilde{Z}}_{e}{\tilde{Z}}_{s}}$$

is the correlation between the residuals of the two regression equations, e.g., 1 – p(“at risk”). Component

(4) $$C_{2}=r_{{\tilde{Y}}_{e}{\tilde{Z}}_{s}}$$

is the correlation between the predicted criterion probability and the residuals of the participant’s regression model. In the final part,

(5) $$C_{3}=r_{{\tilde{Z}}_{e}{\tilde{Y}}_{s}}$$

is the correlation between the predicted probability from the participant’s model and the residuals of the ecological regression model. In the formula (1), the Lens Model Equation correlation indices (G, C_1_, C_2_, and C_3_) are multiplied by the ratios of computed standard deviations of actual values, predicted values and their residuals in either the ecological or the judgement model. The first product can be considered the proportion of the participant’s achievement which is explained by the model.

To investigate each participant’s accuracy, functional achievement was represented by the correlation (r_a_) between the participant’s judgements (*Y*_*s*_) and the true values (*Y*_*e*_) [38]. In the linear Lens Model Equation, the ecological predictability (*R*_*e*_) was derived from the correlation between the true values (*Y*_*e*_) and the predicted values $\left({\hat{Y}}_{e}\right)$ of the ecological model. Cognitive control (*R*_*s*_) was calculated from the correlation between the participant’s judgements (*Y*_*s*_) and the predicted values $\left({\hat{Y}}_{s}\right)$ of the participant’s model. In the logistic Lens Model Equation, the analogous concepts are represented by the ratios of the standard deviation of the model prediction (the category probability) over the standard deviation of the data.

The logistic regression models for the ecology and for many of the participants were associated with large standard errors. The large standard errors in the models suggest that the scenario sample size was inadequate for the logistic regression models, particularly given the degree of cue intercorrelation. To allow all aspects of the Lens Model Equation analysis, including relative cue weights, we chose for this report to ignore the cue that had the highest intercorrelation with the other cues, the patient’s level of consciousness. To produce relative weights, stepwise logistic regression was done for each participant with adjustment of thresholds for retention of predictors - if necessary - until a solution was found in the model where regression coefficients did not have high standard errors. Cues not entered were assigned 0 weight. In this way it was possible to produce all the Lens Model Equation parameters and relative weights for each participant. Regression analyses were conducted using SPSS version 19 (http://www.spss.com).

#### Comparisons of Lens Model Equation parameters

The Lens Model Equation parameters $\left(r_{a},G,\frac{\sigma_{{\tilde{Y}}_{s}}}{\sigma_{Y_{s}}}\mathrm{and}\frac{\sigma_{{\tilde{Z}}_{s}}}{\sigma_{Y_{s}}}\right)$ of nurses’ risk assessments were compared between the paper case simulations and high fidelity physical simulations. Lens Model Equation parameters (r_a_, G) are correlations, and so not normally distributed; Fisher’s z transformation [35] normalizes them, and allows for undertaking Student’s t tests. Wilcoxon matched-pairs signed-ranks test was used to test for the significance of the median difference in the parameters $\left(\frac{\sigma_{{\tilde{Y}}_{s}}}{\sigma_{Y_{s}}}\mathrm{and}\frac{\sigma_{{\tilde{Z}}_{s}}}{\sigma_{Y_{s}}}\right)$ between the paper case simulation and physical simulation conditions. Comparisons of Lens Model Equation parameters were conducted using SPSS and Stata 10 (http://www.stata.com).

#### Relative weights

The logistic regression software provides only unstandardized regression coefficients, so we standardized them using the formula

(6) $$\beta_{i}=B_{i}\frac{SD_{\mathrm{Xi}}}{SD_{Y}}$$

where *B*_*i*_ is the unstandardized coefficient, *β*_*i*_ the standardized, and *SD*_*Xi*_ the standard deviation of cue *i*. Cooksey [26] notes that the standard deviation of the dichotomous dependent variable, *SD*_*Y*_, has questionable meaning. However, the *SD*_*Y*_ term is cancelled out in the next step, normalization

(7) $$\frac{\left|\beta_{i}\right|}{\sum_{j}\left|\beta_{j}\right|}$$

The original sign of the relative weight is restored by multiplying by *β*_*i*_/|*β*_*i*_|, i.e., by 1 or −1, yielding

(8) $$RW_{i}=\frac{\beta_{i}}{\sum_{j}\left|\beta_{j}\right|}$$

It should be noted that with the logistic regression models, the stepwise model used to create relative weights was not necessarily the same as the model used to create the Lens Model Equation parameters, because a model with a non-unique solution can nonetheless produce the predictions needed for Lens Model Equation correlations.

#### Analysis of judgement consistency

Judgement consistency was examined using Phi coefficients on 5 repeated cases drawn from the pool of 25 scenarios. The Phi coefficient [39], a chi-square based measure of association, measures the degree of the association between two binary variables. The Phi coefficient has important advantages over other approaches, e.g., Cohen’s Kappa Statistic [40]. The Phi statistic estimates the chance-independent agreement between nurses’ judgements using data from a 2 × 2 table.

The statistical significance of differences between Phi coefficients was examined by parametric bootstrap simulations on the distribution of differences for two Phi coefficients measures. This was conducted using Stata 10’s bootstrap procedure. Bootstrap standard errors (SE) for the difference of Phi measures between groups were generated using 50 bootstrap replications; between 50 and 200 replications are generally adequate for estimates of bootstrap standard error [41,42].

## Results

### Participants

Ninety-seven (34 experienced and 63 student) UK nurses participated in both the paper case and high fidelity physical simulation arms of the experiment. The majority (n = 27, 81%) of experienced nurses were educated to diploma or first degree level. They had on average 12 years of general clinical experience. Fifty-nine of the 63 nurse students (93.7%) were 2nd and 3rd year undergraduate students and 4 (6.3%) were 1st year postgraduate diploma students. All the students had been in the simulation facilities numerous times for learning activities in the previous study periods and they have completed a range of learning activities, using the simulation facilities, on the topic of “managing the deteriorating patient” – these include, monitoring a patient’s vital signs, clinical risk assessment and cardiopulmonary resuscitation. All experienced nurses confirmed that the high fidelity physical simulation environment was similar to their practice environments.

### Differences between parameters of paper case based and physical simulation based judgements

Table 4 summarizes the Lens Model Equation parameters for judgements of paper cases and high fidelity physical simulations for the Logistic Lens Model Equation. Judgement accuracy with physical simulations (mean r_a_ = 0.502, SD 0.145) was significantly less than with paper case simulations (mean r_a_ = 0.553, SD 0.141; t (96) = 2.74, p = 0.007).^1^ Comparison of Lens Model Equation parameters from the same type of model gives insight into the sources of the better achievement when the participants assessed paper cases. Participants had significantly higher modeled knowledge utilization (G) with the paper case simulations than the high fidelity physical simulations, but were equal in cognitive control $\left(\sigma_{{\tilde{Y}}_{s}}/\sigma_{Y_{s}}\right)$ in both settings. Subgroup analyses showed that both experienced nurses and students had substantially decreased judgement performance in mean r_a_ with physical simulations than with paper case simulations (experienced nurses 0.50 in physical simulations vs. 0.55 in paper case simulations; students 0.50 in physical simulations vs. 0.55 in paper case simulations).

**Table 4:** The logistic regression Lens Model Equation parameters of paper case simulation based judgements and physical simulation based judgements (N = 97)

	**Paper case**		**Physical simulation**		**Difference**
**Logistic regression Lens Model Equation parameters**	**Mean (SD)**	**Median**	**Mean (SD)**	**Median**	**Wilcoxon statistic (p)**
r_a_	0.552 (0.141)	0.577^*#^	0.502 (0.145)	0.503^#^	2.58 (0.01)
G	0.720 (0.126)	0.723^*#^	0.687 (0.141)	0.696^*#^	1.95 (0.051)
C_1_	0.059 (0.189)	0.000^*#^	0.050 (0.165)	0.000^*^	0.32 (0.75)
C_2_	0.010 (0.060)	0.000^*^	0.002 (0.073)	0.000^*#^	1.39 (0.17)
C_3_	0.075 (0.137)	0.056^*#^	0.054 (0.122)	0.029^*#^	1.26 (0.21)
$\sigma_{{\tilde{Y}}_{e}}/\sigma_{Y_{e}}$	0.743 (0)	0.743	0.743 (0)	0.743	
$\sigma_{{\tilde{Z}}_{e}}/\sigma_{Y_{e}}$	0.676 (0)	0.676	0.676 (0)	0.676	
$\sigma_{{\tilde{Y}}_{s}}/\sigma_{Y_{s}}$	0.880 (0.150)	.999^*#^	0.862 (0.173)	0.999^*#^	0.38 (0.70)
$\sigma_{{\tilde{Z}}_{s}}/\sigma_{Y_{s}}$	0.309 (0.340)	0.000^#^	0.326 (0.356)	0.000^#^	1.38 (0.17)

r_a:_ Judgement accuracy; G: Knowledge; C_1_, C_2_, C_3_: Unmodeled knowledge.

Note: Both environmental criterion and judgement are dichotomous. * Skew significantly different from 0; # Kurtosis significantly different from 3.

### Relative weights

In the model predicting the environmental criterion, the respiration rate cue had the greatest importance, with the mean relative weight of respiration rate (0.592) in the logistic regression model with stepwise selection of cues. In predicting the participants’ judgements, the stepwise logistic regression again gave highest weight to respiration rate (mean of 0.573 for the paper case based judgements, and 0.556 for the high fidelity physical simulation based judgements). Of the 97 participants, only 62 participants gave respiration rate the most weight in paper cases whilst 60 participants gave respiration rate the most weight in physical simulations.

### Judgement consistency

Participants’ agreement on the 5 repeated cases was moderately high, with no significant difference between the high fidelity physical simulation assessments (Phi 0.741) and the paper case simulation assessments (Phi 0.777, bootstrap SE 0.023, z = 1.58, P = 0.12).

## Discussion

Our study has addressed whether paper case simulations evoke similar judgements as realistically simulated situations, by comparing nurses’ risk assessments elicited from paper cases with those from physically simulated patients. The findings showed that nurses’ judgements observing patients in the high fidelity physical simulations were significantly less accurate compared with judging paper cases.

Paper cases, whilst relatively easy to administer [10,11,43], may fail to reflect reality and evoke real clinical behavior [44]. Physical simulation, by presenting task information in a way that is perceptually similar to the clinical ecology, allows clinicians to make judgements in settings more similar to their routine clinical environments. Using the technical parlance of judgement analysis, both paper case and physical simulations in this study were identical in their “formal representativeness” [45]. Scenarios in both conditions were sampled from real patient cases in order to retain distributions and inter-correlations among cues participants would ordinarily encounter. However, physical simulations improved substantive representativeness [45]. In our physical simulations, visual displays on standard monitoring equipment including heart rate, systolic blood pressure, temperature readings, respiration rate, together with auditory information such as synchronised breath sounds and moan/vocal sounds regarding different consciousness levels, are simulated to recreate real-life situations, thereby substantially improving the task representativeness in relation to substantive representativeness.

Despite the wide use of paper cases, whether they can elicit judgements akin to those made in the clinical setting is debatable. Some early studies showed that judgements made in response to paper-based scenarios resemble those made with actual patient cases [17,46,47]. For example, Kirwan et al. [46] studied nine British rheumatologists’ judgements on disease severity for outpatients with rheumatoid arthritis. Rheumatologists’ judgements about real patients were significantly correlated with their judgements about the paper-based presentation of the same real life cases some weeks later. Another study by Chaput de Saintonge & Hathaway [17] investigated seven general practitioners’ antibiotic-prescribing judgements for otitis media, using both written information simulations and photographs of ear drums, and reported that written information and photographs evoke similar judgements. However, the generalisability of these studies is compromised by small, self-selected samples of participants drawn only from primary care.

In contrast, a number of studies have found that judgements elicited in paper cases differ from those seen in actual practice settings [44,48]. Morrell & Roland [44] found no significant correlations between general practitioners’ responses to written case vignettes and their actual referral rates, concluding that responses to paper cases might not reflect real clinical behavior. Holmes et al. [48] found physicians order fewer tests in actual practice than with hypothetical paper cases. The findings reviewed by Jones et al. [49] also demonstrated paper cases’ inability to predict actual clinical behavior.

Our study advances these earlier studies in several ways. First, the study recruited a large number of participants, according to *a priori* sample size calculation. Further, the previous judgement analysis studies primarily focused on whether participants gave the same judgements on paper cases and actual practice, thus focusing on only the judgement model. However, our study used records of real patient cases to derive a valid ecological criterion [50]. This allowed it to examine judgement accuracy by comparing an ecological model with models derived from judgements of the paper cases and of the simulated patients.

### Decomposition of judgements

Analysis of the Lens Model Equation decomposition shows that the decrement in judgement accuracy with high fidelity physical simulations compared with paper case simulations is due to the participants having significantly less policy matching. The G parameter, representing matching between models of the judgement and the ecology, is statistically significantly lower in the physical simulation judgements, than in the paper case simulation judgements. There were no differences between paper case and physical simulation models in the other type of parameter, the cognitive control with which knowledge was applied $\left(\sigma_{{\tilde{Y}}_{s}}/\sigma_{Y_{s}}\right)$ in the Logistic Lens Model Equation). Consistent with the latter finding, there were no differences in how consistent the repeated judgements were. It appears that the participants’ judgements were less accurate when judging the simulated patients because they were less able to utilize the vital signs and symptoms in a manner similar to the way those signs relate to the outcome measured in the ecology, as evidenced by the significantly decreased G in physical simulations.

How can we explain why nurses’ accuracy (r_a_) and policy matching (G) in assessing the physically simulated cases are lower than with paper cases? In other studies, unreliable information acquisition from high fidelity cues has prevented participants from achieving high performance [13,51]. In this study, judgement reliability *R*_*s*_ was no different between paper case simulations and physical simulations, thus we can reject explanations focusing on participants’ judgement consistency. Further, if the need to gather information from the monitor or the computerized patient simulator had added variability, this would have led to inconsistency in sticking to one’s judgement policy. Notably, the only significant difference in Lens Model Equation parameters associated with the decreased accuracy judging simulated patients was the nurse’s knowledge G: the degree to which their utilization of cues in making their judgements matches the relation between those cues and the ecological criterion. This could be due to two processes. First, individuals are able to perceive a smaller proportion of the cues relating to the physically simulated patients. Or second, their strategy for integrating the simulation laboratory information could be less related to the way those cues are associated with the outcome in the ecology. The Lens Model Equation analysis *per se* is not able to decompose these processes because its models are “paramorphic” [5]. Our tentative conclusion is that it is through the better utilization of the most important patient information, rather than better acquisition of information or higher consistency of judgment, that the participants made more accurate use of the paper case information than the physical simulation information.

### Limitations

In designing the study, some choices regarding the statistical properties of the set of cases to be judged were made in order to balance several conflicting objectives. To make the task easier so that many nurses would participate, we wanted each to judge a small number of cases. Having few cases would also help avoid boredom, learning, and habituation as a task is repeated, which can alter a participant’s judgement policy [52]. In order for the descriptive models to be generalizable on the basis of the set of cases with realistic cue and criterion distributions, the cases were sampled from a large case series of real patients [28]. However, for statistical efficiency it is better to have the proportion of predicted positive cases ‘at risk’ to be about 50%, particularly when the number of total cases is small. On the other hand, to fit accurate descriptive regression models, particularly logistic models with intercorrelated cues, a large number of patients is required [26,53,54]. In designing any judgement analysis research, it is important to note that compromises are often required between an ideal study design and practical constraints [55]. In this study the number of cases judged was small [26], particularly for logistic regression with high cue intercorrelations [53,54]. Accordingly, the estimated relative weights may be less accurate when entering all cues in the model simultaneously. To address this issue we used stepwise logistic regressions for the analyses of cue relative weights until a unique solution was found in the model where the participant’s regression coefficients did not have high standard errors.

All participants judged the paper cases before the physical simulation cases, which may potentially confound paper-physical task differences because of familiarity effects [52]. We could have randomized the task order, but the fact we did not is unlikely to have influenced participants’ judgements on physical simulations given that participants were not given any feedback regarding the correctness of their paper-based judgements after completing paper cases. Ultimately, any familiarity effect would have led to overachievement in the physical simulations, but in fact participants did less well in this simulated condition. Importantly, to minimize any maturation effect during the time gap between paper case simulations and physical simulations, the physical simulations were conducted shortly after participants completed paper cases.

The high fidelity physical simulation judgement task was designed to be more representative of the situations in which nurses practice than the paper case simulation judgement task. However, in clinical practice a large amount of information is available to nurses assessing risk, including other perceptual cues (e.g. patient’s skin pallor). Whilst such cues may be redundant for predicting critical event risk, the fact that even redundant cues are absent in both physical simulation and paper case scenarios means that actually recognizing the patient ‘at risk’ may be more difficult. Additionally, simulation manikins lack interactivity. This is most clear, perhaps, with the vocal sounds used to represent levels of consciousness. In clinical settings, nurses may assess patients’ level of consciousness by talking to the patient to elicit a response. These features may limit the generalization to real clinical environments of the results derived from judgement studies using simulation laboratories. Despite these limitations, using physical simulation as a vehicle for improving the fidelity (and thus representativeness) of clinical scenarios is a promising approach to eliciting and evaluating clinicians’ reasoning and judgements.

## Conclusions

This study addressed an important methodological question: whether clinician judgements of paper cases, as conventionally employed in judgement analysis research, can be generalized to judgements in clinical situations. To the extent that accuracy in judgement analysis studies is a function of task representativeness, when we improved task representativeness by creating case simulations that were more perceptually representative of the clinic, participants’ performance was affected. They assessed patient risk less accurately in high fidelity physical simulations than when judging paper cases. The use of more perceptually realistic clinical settings (e.g., physical simulations) for assessing nurses’ judgement competency merits scrutiny in future judgement analysis research. Increased awareness of the characteristics of complex and ill-structured tasks may, to some degree, promote the development of interventions to support nurses’ acquisition and integration of clinical information. The paper highlights the importance of using ‘representative’ task environments to elicit clinicians’ realistic judgements. The approach used in this study may be of value to those designing and evaluating clinical simulations as part of education and training strategies aimed at improving clinical judgement and reasoning.

## Endnotes

^1^ For lens model parameter comparisons between paper case simulation and physical simulation based judgements, the same category of statistical significance was obtained using t-tests of the differences in correlations and in Fisher-Z-transformed correlations, and using the Wilcoxon matched pairs signed ranks test. For the G parameter of the logistic lens model, the significances were p = 0.033, 0.052 and 0.051, respectively.

## Competing interests

The authors declare that they have no competing interests.

## Authors’ contributions

HY and CT were responsible for the study conception and design. HY and AF performed the data collection. HY, RH, MB performed the data analysis. HY was responsible for the drafting the manuscript. HY, CT, RH made critical revisions to the paper for important intellectual content. HY, RH, MB provided statistical expertise. All authors read and approved the final manuscript.

## Pre-publication history

The pre-publication history for this paper can be accessed here:

http://www.biomedcentral.com/1472-6947/13/62/prepub

## Supplementary Material

Additional file 1 The clinical vignette questionnaire. Click here for file
