# Supplementary material for: The effect of improving task representativeness on capturing nurses’ risk assessment judgements: a comparison of written case simulations and physical simulations
Source: BMC Med Inform Decis Mak. 2013 May 30;13:62. doi: 10.1186/1472-6947-13-62 (PMC3674950; doi:10.1186/1472-6947-13-62)
Supplement: Additional file 1 — The clinical vignette questionnaire. [file 1472-6947-13-62-S1.doc]

**Additional file 1: The clinical vignette questionnaire**

Clinical information

Mr. Robert Wright, 63 years old and 76 kg weight, was presented to the emergency room in your hospital, accompanied by his wife. He was generally feeling unwell, with a tender abdomen and vomited after each meal for past 2 days. He was born in England and he has been married for 38 years. He is a senior engineer in an automotive company. He has no food or medical allergies. There was no report of use of medications. He has no significant past medical history or history of mental illness. The details of family history are unclear. The following sets of information are available to you when you assess Mr. Wright on admission. Please make your judgements for each scenario.

The sets of scenarios used the following units:

Systolic blood pressure mmHg

Heart rate bpm (beats per minute)

Respiratory rate bpm (breaths per minute)

Temperature °C

An example paper-based patient scenario and response sheet

| Systolic blood pressure 92  Heart rate 92  Respiratory rate 34  Temperature 37.2  Conscious level Reacting to voice | Risk (circle)  YES NO |
| --- | --- |

An example response sheet for the physically simulated patient scenario

| Scenario 1 | Risk (circle)  YES NO |
| --- | --- |
